# Wickersheimer’s Preservative Fluid

**Published:** 1880-04

**Authors:** 


### WICKERSHEIMER'S PRESERVATIVE FLUID.

The *Berliner Klin, Wochenschrift*, (No. 44, 1879,) publishes an official memorandum of the new preparation for preserving dead bodies, animals or plants, which has been invented by Herr Wickersheimer and adopted by the German Government.

The composition of the fluid is as follows:

Alum, 100 grammes, Common Salt, 25 gr; Saltpetre, 12 gr.; Potash, 60 gr. and Arsenions Acid 10 gr., are to be dissolved in 3000 gr. boiling water. On cooling, the liquid is to be filtered. To every 2½ litres, supposing a large quantity to be prepared at once, 1 litre of glycerine and 250 ccm. of methylic alcohol are to be added.

Herr Wickersheimer states that the bodies of animals or men preserved with this fluid retain their form, color and pliability completely. After several years, the muscles look as fresh on section as if they belonged to a recent corpse. For embalming purposes, the body is first injected with the fluid in the proportion of 1½ litres for a child of two years, and of 5 litres for an adult.

It is then immersed in a bath of the fluid for several days, after which it is rubbed dry, swathed in bandages, wetted with the fluid and preserved in an airtight case. For bodies which are to be dissected, the injection alone suffices. Small vertebrates and invertebrates can be kept simply immersed in the fluid, or if wanted in the dry state, may be in it 6 to 12 days and then be taken out and dried in the open air. Hollow organs, such as the lungs and intestinal tract are best injected with it before immersion. The process seems to have the recommendation of simplicity and cheapness, as well as that of preserving the natural color and the pliability of the objects treated by it. It is certain that its merits must have been well tested before receiving the *imprimatur* of the German Government.—Med. Times and Gazette, Dec. 6th, 1879.

At a recent meeting of the Academy of Natural Sciences of Philadelphia, Dr. Leidy stated that this liquid was almost identically the same as that which had been used at the University of Pennsylvania for years, by the late Dr. Horner and himself, and it was simply a modification of the fluid used in the West for the preservation of hams, arsenious acid being substitued for the starch of that mixture.—*The Med. News and Abstract, March,* 1880.

